# Cook County Hospital—A Case of Rupture of the Diaphragm and Stomach

**Published:** 1879-09

**Authors:** J. H. Salisbury


### Clinical Reports.

ARTICLE VI.

COOK COUNTY HOSPITAL.

(Reported by J. H. SALISBURY.)

*A Case of Rupture of the Diaphragm and Stomach.*

A male patient was admitted to Cook County Hospital on the evening of July 22d.

The following was his history previous to admission: While intoxicated, he had been seen to stagger against a lamp-post, and afterward fall off the sidewalk into the gutter, where his left side struck upon the covering of a man-hole.

On admission patient was in great distress, and complained of severe pain in the left side. He vomited several times soon after admission. The matters vomited were dark, looking like coffee-grounds, and evidently contained blood.

On examination, a soft swelling was found in the left hypochondriac region. About the center of this swelling was found an abrasion of the skin about 2 Cm. in length by 1 Cm. in breadth. Around this was a circular red line, enclosing a space about 4 Cm. in diameter. Between the abrasion and this red line the skin appeared natural, although the tissues beneath were swollen. Crepitus was felt, and the finger passed in further than natural, as if the ribs had been crushed in at this point. The patient did not appear to be greatly prostrated, but was very restless, and called constantly for water.

The treatment consisted of morphia to relieve pain, and stimulants. Warm fomentations were applied to the side.

July 23d. — His pulse was 120; his temperature 38°. The vomiting of blood had ceased. He was very restless, although he had slept some. There was no tenderness of the abdomen or chest, except in the immediate vicinity of the injury.

July 24th. — He was still quite restless. Pulse, 120 ; temperature, 37.9°. He had great dyspnœa and cyanosis. He coughed a short, hacking cough, and expectorated a little frothy mucus. Large mucus râles were heard over the whole right lung: metallic tinkling, in the mammary region of the left side. The breathing over the upper part of left lung was good — over the lower part it was indistinct. The percussion resonance was good. There was no tympanitic resonance.

10 a.m. — He was very restless. His sputum was tinged with blood.

4 p.m. — Some tympanitic resonance could be found in left hypochondriac region. Patient seemed a little more comfortable.

7 p.m. — Pulse 100, very feeble; temperature, 37.9°. He died during the night.

The autopsy was made twelve hours after death. The eighth, ninth and tenth ribs were found to be broken. A rupture of the diaphragm was found about 10 Cm. in length, situated behind and to the left of the central tendon. Through this rupture the stomach had been forced into the pleural cavity, so that nearly all of the organ lay in the pleural cavity. A rupture of the stomach was found at the point situated uppermost as the organ lay in the pleural sac. The rent in the stomach would easily admit the passage of two fingers. The contents of the stomach and some blood were effused into the pleural cavity. There was no effusion of the contents of the stomach into the peritoneal cavity. There was no inflammation of the peritoneum nor of the pleura.

